# 3′UTR-located *ALU* Elements: Donors of Potetial miRNA Target Sites and Mediators of Network miRNA-based Regulatory Interactions

**Published:** 2007-01-18

**Authors:** Evelina Daskalova, Vesselin Baev, Ventsislav Rusinov, Ivan Minkov

**Affiliations:** University of Plovdiv, Department of Plant Physiology and Molecular Biology, 24, Tsar Assen St., 4000 Plovdiv, Bulgaria

**Keywords:** retroelements, *Alu* elements, miRNAs, evolution, eukaryotic regulatory networks

## Abstract

Recent research data reveal complex, network-based interactions between mobile elements and regulatory systems of eukaryotic cells. In this article, we focus on regulatory interactions between *Alu* elements and micro RNAs (miRNAs). Our results show that the majority of the *Alu* sequences inserted in 3′UTRs of analyzed human genes carry strong potential target sites for at least 53 different miRNAs. Thus, 3′UTR-located *Alu* elements may play the role of mobile regulatory modules that supply binding sites for miRNA regulation. Their abundance and ability to distribute a set of certain miRNA target sites may have an important role in establishment, extension, network organization, and, as we suppose – in the regulation and environment-dependent activation/inactivation of some elements of the miRNA regulatory system, as well as for a larger scale RNA-based regulatory interactions. The *Alu*-miRNA connection may be crucial especially for the primate/human evolution.

## Introduction

In many aspects, the eukaryotic cells, including our own, still live in a RNA world. Genomes of most multicellular eukaryotes are flooded with mobile elements, especially retroelements – products of 3–4 billion years of activity of an ancient enzyme reverse transcriptase [7]. Related to this phenomenon is another one: the abundance of non-protein coding RNA transcripts, many of them being functional participants in a hypercomplex RNA-based regulatory networks [8, 10]. Revealing the numerous and complex connections existing between these two RNA-related phenomena could be crucial for the understanding of modern life and evolution.

In this article we focus on the connection of a specific group or mobile elements *– Alu* elements to miRNA regulation.

### Mobile elements—agents of permanent change

Mobile elements are segments of DNA that can move to different positions in the genome of a single cell. In this process, called transposition, they may cause a substantial range of changes in DNA sequences (from point mutations to large scale recombinations). They also may increase (or decrease) the amount of DNA in the genome [11–14].

There are two (in some classifications – three) distinct types of mobile elements [12, 14]:

#### Class II – DNA Transposons

They consist only of DNA that moves directly from place to place. Most of the DNA transposons move by a ‘cut and paste’ process: the transposon is cut out of its location and pasted in another. This process requires a transposase, an enzyme encoded within some of DNA transposons. Often transposons have lost their transposase genes (they have become non-autonomous) but still can transpose with the help of enzymes encoded by other DNA transposons.

After sequencing of many genomes, some authors distinguish a third class of mobile elements:

#### Class III Transposons or Miniature Inverted-repeats Transposable Elements or MITEs

MITEs are too small to encode any protein, so probably they also make use of enzymes from larger transposons. MITE elements often reside near or within genes, so they might have some useful role there, i.e. they are ‘genetic symbionts’ [15].

#### Class I – Retroelements

As the main subject of our research is the *Alu* element (a SINE retroelement), we discuss this group in more detail below.

Retroelements use reverse transcriptase (RT) to make a DNA copy of their RNA transcript and then insert it in a new location. The mode of retroelements’ transposition is ‘copy and paste’, so they generate numerous copies and appear to be the main reason for expansion of many eukaryotic genomes. About 40% of the entire human genome consists of discernible retroelements, and perhaps more than other 40% of non-protein coding DNA is made of ancient retroelement copies that have accumulated many mutations and became indiscernible [6, 7]. Main classes of retroelements are: **LTR-retroelements,** flanked by long terminal repeats (LTRs) at their ends, and **non- LTR-retroelements,** separated on two main sub-groups:

##### LINEs (Long interspersed elements)

The human genome contains more than 850,000 LINEs (about 21% of it). Most of them belong to LINE-1 (L1) family. L1s are about 6,500 bp long and encode an endonuclease and a reverse transcriptase. L1 elements are the main producers of retropseudogenes, of functional retrogenes, and of non-authonomous SINE retroelements [11, 12, 14].

##### SINEs (Short interspersed elements)

SINEs are short DNA sequences (100–400 base pairs). They represent reverse-transcribed small non-protein coding RNA molecules originally transcribed by RNA polymerase III (tRNA, 5S rRNA, 7SL RNA etc.) [3, 4, 6, 11, 14]

The most abundant SINEs are the ***Alu*** **elements.** They are restricted to primate lineage. There are over a million *Alu* copies in the human genome, representing about 11% of the total DNA. *Alu* elements are about 300 bp reverse transcripts of the 7SL RNA, the RNA part of the signal recognition particle. Being non-autonomous, *Alu* elements use the autonomous L1 family of LINE retroelements (long interspersed elements) as their “transporters”. The *Alu* sequence has dimeric organization and its left monomer carries functional sequences of RNA Pol III promoter. *Alu* elements contain also cryptic splicing sites and many other regulatory and regulatory-like motifs that can influence the gene expression in various ways. [11–14]

During the past 65 million years, *Alu* elements have propagated to more than one million copies in primate genomes, which have resulted in the generation of a series of *Alu* subfamilies and sub-subfamilies. There are three *Alu* subfamilies, *Alu* J (oldest), *Alu* S (intermediate age), and *Alu* Y (youngest), divided on the base of their evolutionary age. These subfamilies are further classified into sub-subfamilies based on their divergence from consensus sequence [3].

Generally, the autonomous and non-autonomous mobile elements have different behavior to genes. The autonomous elements usually insert away from gene-rich areas, while the non-autonomous elements, including *Alus*, often insert near and even within the genes. The ‘*Alu* transporters’ – autonomous L1 elements, preferentially insert in gene poor genome positions; in contrast, they often insert *Alu* elements in close proximity and even within protein coding genes. Like MITEs, *Alu* elements are thought to be generally genetic symbionts, although their insertions often cause mutations and diseases. Human hnRNAs are often found to contain *Alu* elements; most of them in introns, but also in UTRs and even exons (2% of *Alu* inserts). Interestingly, among the UTR-located insertions, there is strong preference to 3′UTRs (84%) compared to 5′UTRs (14%) [3, 4, 13].

### Mobile elements in evolution and regulation of gene expression

The abundance and sequence similarity of mobile elements’ copies make them triggering force of all scales of genome reshaping events. Mobile elements are the main cause for genome plasticity, which, although often deleterious for individual organisms, in evolutionary means appear to be a “genomic treasure” - a main driving force of genome evolution. [7, 8, 11, 12, 14]

Mobile elements have also various and deep impacts on establishment, function and evolution of cellular regulatory systems. Below we discuss in brief some of the most important effects of mobile elements on the gene regulation.

#### Mobile elements – donors of ready-to-use regulatory motifs

Various mobile elements are found to carry almost all known regulatory elements:

- promoters [11, 13]
- RNA polymerase II enhancers [11].
- splicing sites [11, 19]
- Pol II Transcription-Modulating Elements (TMEs) and other transcription regulatory elements [11, 13, 14]
- polyadenilation signals [11, 13, 14] etc.

The sequence similarity within the groups of mobile elements and ‘copy and paste’ mode of transposition may have contributed to broader distribution and network propagation of such regulatory motifs in genomes.

#### Mobile elements in the emergence and the evolution of epigenetic regulatory systems

##### Mobile elements in the DNA methylation and imprinting

It is thought that DNA methylation initially emerged as genomic defense mechanism against invasion of mobile elements. Then, insertions of mobile elements near and within genes may have facilitated the “switch” of this epigenetic mechanism from transposons to cellular genes. [3, 18, 19, 20] CpG islands, residing within *Alu*s are often differentially methylated, sometimes in parental-dependent manner – a link of *Alu* elements to the phenomenon of genomic imprinting. [3].

##### Mobile elements and the histone modifications

Recently a novel link was discovered between histone modifications and activity of mobile elements. A specific histone deacethylase in human genome is engaged with control of transposons [16].

##### Mobile elements and the alternative splicing

*Alu* elements inserted in UTRs and CDS often contain splicing sites, thus mediating the alternative splicing and it may be a widespread phenomenon. [3, 4, 19, 20]

##### Mobile elements and RNA editing

The transcripts of mobile elements, especially *Alus* are often subjects to intensive and specific A to I editing [17, 23].

##### Mobile elements and srRNA-based regulation (RNA interference and miRNA regulation)

Recent research revealed at least two complex regulatory systems based on so called small regulatory RNAs (srRNAs). **RNA interference (RNAi)** is a srRNA-based system of posttranscriptional silencing [34, 35]. The active small RNA molecules guiding RNAi are called small interfering RNAs (siRNAs) and are produced by cleavage of long dsRNA transcripts of various origins into 20–22 nt molecules.

The RNAi and miRNA regulatory systems are different, but appear to be closely related. **MicroRNAs (miRNAs)** are evolutionarily conserved small non-protein-coding RNA transcripts that regulate gene expression at the post-transcriptional level [27–36]. They appear to be key regulators of eukaryotic gene expression, yet the question of how microRNA expression is itself controlled remains unclear. In animals, mature miRNAs are ~22 nucleotides long and are generated from a primary transcript (termed pri-miRNA) through sequential processing by nucleases belonging to the RNAseIII family. The first of these enzymes, Drosha, cleaves the pri-miRNA and excises a stem-loop precursor of ~70 nucleotides (termed pre-miRNA), which is then cleaved by the enzyme Dicer. The endoribonuclease Dicer produces both types of srRNAs: siRNAs and miRNAs. In animals, siRNAs direct the cleavage of target mRNAs, reducing their cellular concentration, whereas miRNAs repress the translation of target mRNAs into protein. Both siRNAs and miRNAs incorporate into similar protein complexes (RNA-induced silencing complex and miRNP complex respectively) and guide it to target sequences [37]. A critical determinant of mRNA cleavage/translation repression is the degree of sequence complementarity between the srRNA and its mRNA target. miRNAs complete their function by base pairing with partially complementary target sites, located in most cases in the 3′UTRs of mRNAs. There is set of requirements to certain sequence to be a functional miRNA target site [2].

An evolutionary model with ideas similar to these about the origin of methylation is proposed for the establishment of the RNAi-based “genome immune system” [48]. It is thought that it has emerged in order to defend the genomes from viruses, mobile elements and defective mRNA transcripts. In *C. elegans* mutations in genes involved in RNAi lead to increase of the rate of transposition.

Apart from the role of mobile elements in RNAi, recently some facts reveal a relation of mobile elements to miRNA regulatory system. It is shown that some miRNA genes originate from genomic L2 and MIR retroelements which also contribute to establishment of target sites [5].

**Figure 1:**
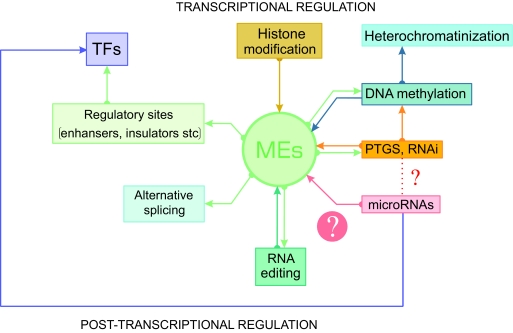
Mobile elements (MEs) and their relation to cellular regulatory processes. The connection between ret-roelements and miRNAs is the objective of current study.

If we summarize all above-mentioned facts, it is clear that mobile elements have almost unlimited regulatory potential. Therefore, it is not surprising that practically all recent cellular regulatory systems are related to them in one way or another. (Figure 1).

Recent investigations suggest that previous estimations of the number of human miRNA genes were low, and that miRNAs regulate at least 20% (and perhaps up to 50%) of human genes [34]. The predicted abundance of miRNA regulation and recent discoveries of involvement of L2 and MIR elements in miRNA regulation, as well as the wide regulatory potential of *Alu* elements, directed us to analyze their sequences for possible relationships to the miRNA regulatory network. Another promising implementation for such interaction might be the affinity *of Alus* to gene-rich regions and the preference to *Alu* insertions in human genome for the 3′UTRs. So we tested **the possibility** ***Alu*** **sequences, inserted in 3**′**UTRs of various genes, to be donors of miRNA target sites.** The emerging picture appears to be very interesting and complex, so in conclusion we suggest another possible function of *Alu* elements in miRNA- and other types of RNA based regulation.

## Materials and Methods

Consensus *Alu* sequences we downloaded from the **Repbase Update** database (http://www.girinst.org) [44]. We retrieved the sequences of all sub-subfamilies available in the database (32-consensus sequences total):

- *Alu* J sub-family: ***Alu*Jo,** ***Alu*Jb** (2 sequences)
- *Alu* S sub-family: *Alu*Sc, *Alu*Sg, *Alu*Sp, *Alu*Sq, *Alu*Sx, *Alu*Sz (6 sequences)
- *Alu* Y sub-family: ***Alu*Y,** ***Alu*Ya1,** *Alu*Ya4, ***Alu*Ya5,** *Alu*Ya8, *Alu*Yb3a1, ***Alu*Yb3a2,** ***Alu*Yb8,** *Alu*Yb9, ***Alu*Ybc3a,** ***Alu*Yc1,** *Alu*Yc2*, Alu*Yc5, ***Alu*Yd2,** *Alu*Yd3, ***Alu*Yd3a1,** *Alu*Yd8, *Alu*Ye2, *Alu*Ye5, *Alu*Yf1, *Alu*Yf2, *Alu*Yg6, *Alu*Yh9, *Alu*Yi6 (24 sequences)

(The 22 sub-sub families of *Alu* elements that we found inserted in 3′UTRs of analyzed genes are shown in **bold**.)

Then we performed 32 BLAST searches using the 32 consensus *Alu* sequences as a queries against the **Refseq_RNA** database at NCBI in order to quickly “catch” *Alu*-containing mRNAs among the ~30 000 human genes. Using BLAST also allowed us to choose *Alu* insertions that are not much diverged from the consensus sequence. We retrieved only the genes with known function and among the best 100 hits from each of the 32 searches. BLAST and NSBI Nucleotide (http://www.ncbi.nlm.nih.gov/entrez) data also helped us to choose transcripts, which have *Alu* insertions in their 3′ UTRs.

BLAST searches were performed using the BLAST search engine at NCBI (http://www.ncbi.nlm.nih.gov/BLAST). We used BLASTN method (nucleotide-nucleotide BLAST, version 2.2.11).

Using BLAST, we collected an initial set of 239 genes with known or strongly predicted functions, having at least one *Alu* insertion in their 3′UTRs. 3′UTRs of all genes, selected for further analysis were obtained from the **Nucleotide** database at NCBI (http://www.ncbi.nlm.nih.gov/entrez/Nucleotide).

Then we used the specialized repeat-searching software **CENSOR** (http://www.girinst.org/CENSOR) [44] to obtain more accurate information about the type, number and localization of *Alu* elements and other concomitant repeats.

Functions and processes in which the analyzed genes were involved were defined using the **Gene Ontology and Annotation (GOA)** recourse (http://www.ebi.ac.uk/GOA). From the Function/process/component tables we selected the basic **keywords** characterizing main areas of activity of the proteins and used them in the functional analyses.

For the **statistical evaluation of results** we used two Web-based tools generating random sequences. We used the **DNA Shuffler** program (http://www.bioinformatics.vg/sms/shuffle_dna.html) to shuffle randomly nucleotides in all analyzed sequences. RSA-tools package (http://rsat.ulb.ac.be/rsat/random-seq_form.cgi) was used to generate random sequences with given length and nucleotide background. The program **CreateBackgroundModel** from the **MotifScanner** package (http://homes.esat.kuleuven.be/~dna/BioI/Software.html) [50] was used to calculate the nucleotide background of consensus *Alu* sequences when generating random sequences. Statistical calculations were made using the MS Excel properties. Supplementary data are stored in .xls format and are available for discussion.

Supporting information about miRNAs and their targets we have obtained from the following databases: **TarBase** – a database of experimentally proved miRNA targets (http://www.diana.pcbi.upenn.edu/tarbase.html) [47], **MiRBase** (http://microrna.sanger.ac.uk/) [45] and **miRNAMap** (http://mirnamap.mbc.nctu.edu.tw/) [46].

In order to find micro RNA target sites, we analyzed the 3′UTRs of selected genes with a **new software tool for prediction of miRNA target sites** called **MicroInspector,** created recently by Ventsi Rusinov and Vesselin Baev [1]. It is a new generation program for miRNA binding sites prediction. It predicts the possible target sites by combining calculation of the free energy and recovering the structure of the miRNA-target duplex. There is a possibility to select a folding temperature, natural for the analyzed organism, and to choose maximum threshold of free energy. Analyzing human genes, these variable parameters were set to 37°C and −23 kcal/mol respectively. In order to select only the duplex structures that match all criteria of a functional target site, we created an additional script that filters the results, and then again inspected all the results manually. The same search procedures were executed for all analyzed mRNAs, shuffled and random sequences.

The **MicroIncpector** tool is available on the Web at the site http://www.imbb.forth.gr/microinspector/.

## Results and Discussion

### Description of *Alu* sequences, inserted in 3′UTRs and found miRNA target sites

Our initial set includes 239 genes with known functions/processes and with at least one *Alu* insertion in their 3′UTR. All genes were analyzed with **CENSOR** to find and unambiguously recognize all *Alus* and other mobile elements they contain. Then we inspected them for presence of miRNA targets with the **MicroInspector** software [1].

CENSOR searches showed that analyzed 3′UTRs are quite densely populated with *Alu*s and other mobile elements. They contain 383 *Alu* inserts and 249 insertions of various other elements (L1 fragments, LTR-elements, MER, MIR, and SVA etc.). The total length of all *Alu* inserts in 3′UTRs is 102543 NT, which represent 19, 78% of the total length of the 3′UTRs (518360 nt). There are 24 genes in which *Alu* insertions contributed more than 50% of the UTR length. On the extreme are the 3′UTRs of the genes ZNF91 (NM_003430), ZNF669 (NM_024804) and NFKBIL2 (NM_013432) which 3′UTRs are practically completely *Alu*-made (*Alu* insertions occupy about 90% of their length). Interestingly, all these 3 proteins are involved in transcription regulation – two zinc finger proteins are predicted transcription factors, and NFKBIL2 is a transcription corepressor.

The ratio *Alu* inserts/gene is 1,6025 – there are many proteins (95, or 39,75% of all genes analyzed) that have more than 1 *Alu* insertion in their 3′UTR. The record here belongs to BIRC4 (NM_001167) and ZNF490 (NM_020714) genes. The first, containing 8 *Alu* inserts is involved in apoptosis and protein ubiqutinization, and the second, containing 6 *Alu* inserts, is a transcription factor.

The CENSOR results about the number and distribution of repeats are interesting by themselves, but the big surprise came when we analyzed the 239 3′UTRs with the **MicroInspector** tool.

**Figure 2:**
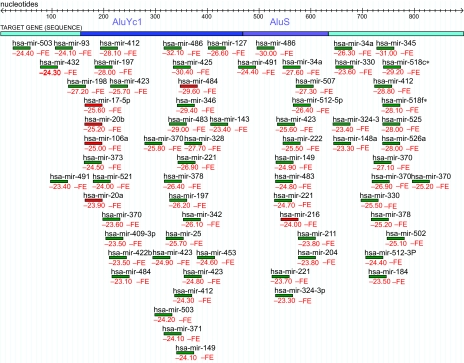
Distribution of target sites on the 3′UTR of the protein chemokine ligand 5 (NM_002985). It carries 2 *Alu* insertions in direct orientation: *AluYc1* and *Alu*S that have occupied 53,5% f the 3′UTR length. All six probable target sites with proper structure (red lines) map in the two *Alu* inserts. All the rest are ‘cryptic target sites’ with sequence complementarity to relevant miRNAs but without proper structure (green lines).

**MicroInspector** predicted presence of miRNA target sites in 3′UTRs of practically all inspected genes (238 of 239). We counted as ‘localized in *Alu* inserts’ all target sites which start position (reported by **MicroInspector**) lies between the start point and 20 nt before the end point of the mobile element (reported by **CENSOR**). The contribution of *Alu* sequences to these numerous targets was quite significant. Only 11 genes did not have miRNA targetsites in their *Alu* insertions (nevertheless they have at least one target site in their 3′UTR outside the *Alu*). 38 of the 238 genes (15,96%) have miRNA target sites **only** in the *Alu* insertions. (Figure 2)

Initially analyzing all UTRs with the **MicroInspector,** we have found 2359 sites total, for 269 different miRNAs. Thus, predicted density of miRNA sites was 1 site on every 219,74 bases, or about 4,54 target sites/Kb. To evaluate the statistical significance of our results we shuffled randomly the nucleotides of all the 239 UTR sequences. Shuffled sequences are suitable for statistical evaluation because they have the same nucleotide background and length as original sequences, but are random. The MicroInspector found in shuffled sequences 1095 miRNA target sites, which means one site at every 473,38 bases, or 2,11 target sites/Kb.

Then we calculated the **occurrence ratio, OR** (number of occurrences of each miRNA target site, divided on total number of proteins) for both the original and the shuffled sequences. All miRNA target sites that have **ORshuffed** ≥ **OR3**_′_**UTRs** were removed as insignificant.

This procedure was performed for all 3′UTRs. Additionally, we made one more statistical clearance only for *Alu* inserts. We generated 383 random sequences, each 268 nt long (268 nt is the average length of *Alu* inserts in the 3′UTRs) and with the same nucleotide background as the sum of 32 consensus *Alu* sequences. Again we calculated the occurrence ratio and removed all results that have lower or equal OR in real *Alu* inserts than in random sequences.

After removing the insignificant hits, there were still many target sites remaining. 3′UTRs as a whole contain 1980 target sites for 153 miRNAs. Of them, 1095 sites for 53 miRNAs are localized in the *Alu* inserts. Of the 1095 miRNA sites localized in *Alu* inserts, 660 (60,3%) occur in *Alu* inserts with direct (sense) orientation and 435 (39,7%) are in inserts with complementary (antisense) orientation.

Thus, it appears that *Alu* elements have contributed to 3′UTRs 55,3% of their miRNA target sites. The miRNA target site density in 3′UTRs as a whole is one miRNA target site at every 262 nt, or about 3,8 target sites/Kb. The miRNA target site density in *Alu* inserts is remarkably high (about 3 times higher than in 3′UTRs as a whole) – one miRNA target site at every 93,7 nt, or about 10,67 sites/Kb.

Moreover, the analyzed 3′UTRs contain 78 significant target sites localized in other mobile elements: various types of LTR elements, L1 fragments, L2, MIR, SVA and MER elements. They contain target sites for 56 different target sites for 56 different miRNAs, and are resided in 3′UTRs of 37 of the genes. In the present ‘*Alu*-centered’ study we regard these sites as localized outside of *Alu* insertions, namely, as a part of the total miRNA target site content of the 3′UTRs, but they could be a subject of separate analysis in the future.

The micro RNA sites that occur 5 or more times *in Alus* are listed in Table 1.

All predicted sites have low free energy, which means they represent stable miRNA/mRNA duplexes. In some cases the free energy is extremely low, the record −42,30 kcal/mol is for a target site for hsa-mir-339 (almost perfect complementary miRNA/mRNA duplex, localized in L2B element). Over 75% of target sites localized in *Alu* inserts have free energy lower than −25 kcal/mol, over 15% - lower than −30 kcal/mol.

**Figure 3:**
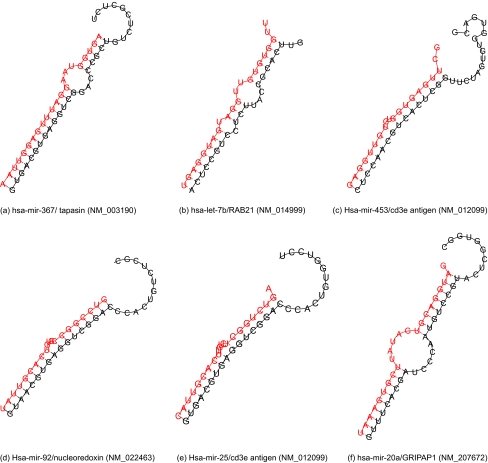

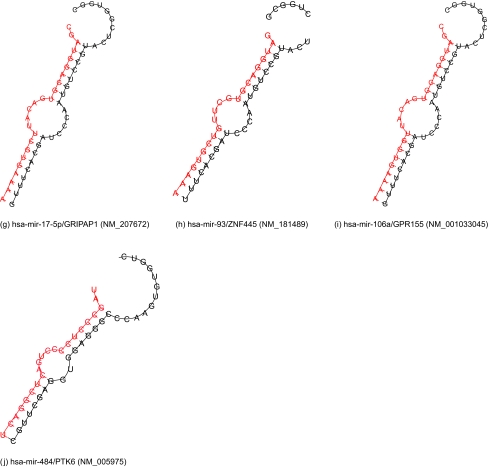
Structures of some miRNA/mRNA duplexes at target sites localized in *Alu* insertions. 3′ end of miRNA is shown at the top of the figures, 5′—at the bottom. miRNAs are colored in red, mRNA targets – in black. (a) – (e) – target sites in antisense *Alu* insertions. over page: (f) – (j) target sites in sense *Alu* insertions.

All target sites, in and out, of *Alu* insertions reported here have a secondary structures characteristic for a functional miRNA/target duplex (Figure 3). All sites reported here have at least 7 nt ‘seed’ complementary region at the 5′ region of the site with maximum 2 G:U pairs; and at least 4 complementary nucleotides at 3′ region.

Such sites match all criteria for a functional target site, described in [2]. According to classification given in the same source, most of the target sites are of canonical type. There are also some typical 3′ compensatory sites and others that are closer to 5′ seed (not shown) but they are rare.

The miRNA target sites in *Alu*s naturally divide in three major categories according to their abundance and distribution:

- **widespread target sites**. They occur in almost all full length *Alu* inserts of relevant orientation. For the *Alu*s in direct (sense) orientation these are represented by the sites for the family of related miRNAs hsa-mir-20a, -20b, -106a, -106b and -93. A hallmark for the *Alus* in complementary (antisense) orientation is the set of sites for the family of hsa-mir-367the set of sites, -92 and -25. As a rule, these target sites almost do not occur outside the *Alu* inserts. (Table 1) These widespread target sites are represented in all *Alu* sub-subfamilies in relevant orientation inserted in 3′UTRs.
- miRNA target sites with **intermediate** levels of occurrence in *Alu* inserts. The target sites for hsa-let-7b, let-7i, let-7c, hsa-mir-484, -453, -346, -422a and some other target sites (Table 1) fall into this category. Intermediate sites are also more abundant in *Alu* inserts than out of them, but also occur, at frequency higher than of the 1^st^ group, in the other parts or 3′UTRs.
- miRNA target sites with **low** frequency in *Alu* inserts (not shown in Table 1). They occur 1–4 times in *Alu* insertions but (most of them) have much higher frequency of occurrence in other parts of the 3′UTRs outside the *Alu* insertions.

This distribution of *Alu-r*elated miRNA target sites is interesting because it may imply some insights about the network relationships in the miRNA-based regulatory pathways. The widespread target sites of the first group, if proved functional, may be crucial in processes as stress response and quick morphological and/or evolutionary transitions, where many proteins with various functions have to be repressed/activated at a same time. In the case with the second group of ‘more individualized’ *Alu-*related target sites, insertions of *Alu* elements may have caused expansion of existing miRNA regulatory networks, adding new members to them. The same possibility, but in more restricted scale, may have happened to *Alu*-related target sites from the third group. The very existence of these three groups indicates that *Alu* elements could play not only the role of distributors of identical target sites to various otherwise unrelated proteins, but they could also cause individual, protein-specific changes.

Besides all these considerations, there is a very important question remaining: why are there so many miRNA target sites? Having in mind the strict selection system we used (only the sites matching all criteria for a functional target sites were selected) and the additional manual inspection of the results, we consider not very probable that the abundance of miRNA target sites is due to the hypersensitivity of the **MicroInspector** program (many false-positives). More probably, 3′UTRs (and perhaps other parts of mRNAs and other genome entities) have high potential of generation of miRNA target sites. This may be due to some kind of evolutionary relationship between sequences of miRNAs and mRNAs. We still don’t now whether (and if yes, how exactly) the origin and evolution of miRNA genes and the origin and evolution of mRNAs are related. Another explanation may be that miRNA based regulation is far more abundant than we expected, and to great extent this is due to mobile elements including *Alu*s. SINEs and other elements may have spread continuously miRNA target sites among mRNAs during evolution. As it is proved in [5], mobile elements could also play a main role in the emergence and distribution of miRNA genes. Further analyses are needed to explore the real magnitude and meaning of these phenomena.

### Origin of *Alu*-localized miRNA target sites

How and when did all these target sites appear in the *Alu* insertions?

All the *Alu* elements in primate genomes originate from retrotransposed copies of a single noncoding RNA – the small cytoplasm RNA, component of signal recognition particle (SRP) – 7SL RNA. In the Genbank database, we found 2 genes and 6 pseudogenes of the human 7SL RNA.

We tested the presence of miRNA target sites in the two 7SL RNA genes with the use of the **MicroInspector** program. In sense orientation they showed no significant presence of miRNA target sites, but in antisense orientation they, like *Alu* inserts, also have the widespread target site for the hsa-miR-367/25/92 family.

With exception of the mentioned sites, all the rest miRNA target sites found in *Alu* insertions in the 3′UTRs sites are not present in the 7SL sequence. It means that they are generated during the initial transcription of the *Alu* by the polymerase III [3], or, what is more probable, during the reverse transcription of the *Alu* sequence by the L1-RT, which is known to be a process generating many mutations.

Nevertheless, the 7 SL RNA, as well as *Alu* sequences, contains many miRNA target sites with small differences from the proper structure but still with a high degree of sequence complementarity to some miRNAs (hsa-miR-187, 151, 210, 217 and 328), i.e. some kind of ‘cryptic’ miRNA target sites. Such cryptic sites abound in *Alu* insertions in analyzed 3′UTRs too (Figure 2). Perhaps in many cases a few mutations are enough to ‘switch on’ these sites (to make them functional). This should be the way for generation of many insert-specific miRNA target sites in *Alu* sequences (groups 2 and 3). The high regulatory potential of the 7SL RNA sequence could be explained with the fact that it is itself a noncoding RNA sequence (an entity from the ancient RNA world), with probable potential of performing many RNA-protein and RNA-RNA interactions.

Then we tested consensus sequences of the three subfamilies (oldest *AlyJ*, intermediate *AluS* and youngest *AluY* ) to see if they have miRNA target sites. They showed presence of all widespread target sites and about 50% of other sites, including let-7b and let-7c site. It means that these sites are conserved across all *Alu* subfamilies, for more than 55 million years of evolution of *the Alu* sequences. This could be another implication for their functionality.

BLAST searches and CENSOR inspections revealed no homologous *Alu* insertions in chimpanzee orthologous mRNAs. Instead, there were some other, nonhomologous chimp genes containing *Alu* insertions, but they were much rare than human genes. This may be due partly to less advanced annotation process of the chimp genome (many of the mRNAs have status ‘predicted’, and in many cases only the coding sequence (without UTRs) is deposited in Refseq_RNA database). Nevertheless it is probable that the majority of UTR-located *Alu* insertions, even these *of Alus* of older subfamilies, to be species-specific. Further comparative studies are needed to reveal this problem in more details.

## Functional relationships

### GOA keywords-based characterization of genes

Based on **GOA Function-Process-Component** system we extracted main keywords assigned to analyzed proteins and used them to specify some functional categories (Table 2). We counted all the keywords and selected for analysis all that occur more than 10 times and obtained 13 categories, including proteins of related functions and/or expression: ‘metabolism’, ‘signal transduction’, ‘transport’, ‘regulation of transcription’, ‘development’, ‘immune response’, ‘receptor’, ‘nervous system’, ‘cell cycle’, ‘protein modification’, ‘structural component’, ‘apoptosis’ and ‘cell adhesion’.

Additionally we decided to include one more group of 8 proteins, matching the term ‘information processing’ (In fact, this is not a GOA keyword but we used it to indicate genes involved in DNA replication and repair, transcription (excluding TFs and other regulators of transcription, which are in separate category), translation, mRNA processing and splicing.

The sum of proteins exceeds 238, and the sum of percents exceeds 100, because some of the proteins match 2 or more keywords simultaneously.

The functional distribution shown on Table 2 partially confirms the observations in [21] that mRNAs carrying *Alu* insertions predominantly encode proteins involved in metabolism, transport and signaling. But in our dataset we can also see a significant proportion of transcription regulators, proteins involved in development (including 4 homeobox genes), cell cycle and apoptosis. All these groups of proteins are among the known and predicted targets in the miRNA-based developmental timing and other miRNA-based regulatory processes. On the other hand, some of the recently proved and/or predicted miRNA targets include transport proteins as solute carriers; receptors and others involved in signaling and cell-cell interactions [46], well represented in our set of genes.

As we can see in Table 2, some of the categories contain more than average miRNA target sites. The greatest number of target sites/protein is observed in genes involved in development, regulation of transcription and cell cycle. Below average is the number of target sites in structural components and genes involved in metabolism and information processing.

The contribution of *Alu* insertions to the miRNA site content in the different categories is different. Most significant is the contribution of *Alu* inserts in the categories ‘cell cycle’, ‘cell adhesion’ and ‘information processing’. The last fact is itself very interesting, as the 8 proteins in this category are involved in housekeeping information processes, previously thought to be ‘forbidden’ for mobile element insertions (this is true also for the homeobox genes, which are represented in our dataset too). The contribution of *Alus* to miRNA-based regulation in genes related to nervous system is also considerable. This fact is very important in the light of the idea that *Alu* elements may have contributed to establishment of some human-specific characteristics.

### Different gene categories have different miRNA target content

As it is expected, the distribution of miRNA target sites between categories of genes is non-homogenous. Certain groups are enriched in certain miRNA sites and decreased in others. In some cases we could predict, on the base of their distribution among categories, the specific function for some of the miRNAs with target sites in or out of the *Alu* insertions.

Because the above groups represent relatively small populations, we performed once again statistical clearance of the matches that appear insignificant for a certain group. We used the shuffled sequences of 3′UTRs from each category to find miRNA target sites that occur at higher or equal frequency to the target sites in the 3′UTRs in that particular category. This way we calculated the occurrence ratios (OR) dividing the number of relevant target site to the number of proteins in the particular category. Then we calculated ΔOR - the difference between OR for each category and OR for all analyzed proteins, OR_total:_

$$\Delta \text{OR}=({\text{OR}}_{\text{group}}-{\text{OR}}_{\text{shuffled}})-{\text{OR}}_{\text{total}}$$

All miRNA target sites that have ΔOR ≥ 0,05 are accounted as significantly enriched in the relevant group; the sites with ΔOR≤ −0,05 are accounted as significantly decreased. The observation of the enrichment/diminution in a certain category is more useful when certain miRNA has many targets, as it is the case with the widespread *Alu*-localized sites; cases of ‘individual relationships’ (a miRNA having 1–2 targets) could hardly be detected in this system. An additional obstacle comes from the fact that we are not sure what the functional range of targets of miRNAs is. So we tried to find keywords general enough to cover a biological process, and at the same time specific enough to discern between different functional categories. We made some interesting observations about the distribution of different target sites among different categories of genes. On this base, we made also some assumptions about putative functions of some miRNAs. Some of our conclusions are directly or indirectly supported by the **TarBase**, and indirectly from MiRanda target site predictions reported in the **miRNAMap** site.

#### A) Distribution of the widespread target sites

The category of genes matching the keyword ‘development’ is significantly enriched in antisense (complementary) *Alu*-localized target sites for **hsa-mir-367, -25, and 92,** and at the same time shows decrease in sites localized in sense (direct) *Alu* insertions as **hsa-mir-93, -17-5p, -20, -106.** This is interesting because is an opposition of the general distribution in all proteins (roughly 60:40) (60% sites in direct *Alu* insertions: 40% sites in complementary *Alu* insertions). Such “reverse” distribution is observed also in categories ‘cell cycle’, ‘transport’, and, to lesser extent, ‘transcription regulation’ and ‘immune response’. The sites in *Alu*s with sense orientation are overrepresented most significantly in the categories ‘information processing’ (the sites for **hsa-mir-106a** and -**20b** occur in 8 of 7 proteins in this category), and ‘protein modifications”, where 12 of 15 proteins have **hsa-mir-17-5p** site and 11 of 15 – **hsa-mir-20b** site. The enrichment in these sites in categories ‘apoptosis’, ‘cell adhesion’, and in the proteins expressed in nervous system is also considerable. Categories ‘signal transduction’ and ‘metabolism’ contains sense and antisense inserts-localized target sites in proportion similar to the general.

The existence of all miRNAs that target widespread sites in *Alu* insertions is experimentally validated in human cells by cloning and/or Northern analysis. A common problem when investigating miRNAs is that their targets, especially in animals and human, is much harder to be found and proved than the miRNAs themselves. There are no experimentally validated targets in the databases for the hsa-mir-367, -25, and 92. **MiRNAMap** reports some target sites for them, predicted by MiRanda program: the RPA-binding transcription activator, the transmembrane protein TED and the putative chap-erone DNAJB12 for hsa-miR-92 and 25; MiRanda also predicts 3 mir-367 targets: the neuronal membrane glycoprotein M6A and round spermatid basic protein 1 – both with unknown function, and a potential phospho-lipids transporting ATPase. These predictions indirectly confirm the involvement of above mentioned miRNAs in transcription regulation and transport, presumed on the base of the distribution of their target sites in antisense *Alu* transcripts in our research.

A little more is known about the targets for hsa-mir-93, -17-5p, -20, -106. There are some experimentally validated targets for these miRNAs. In **TarBase**, there are two validated targets for hsa-mir-20 and one for hsa-mir-106a. One of the hsa-mir-20 targets is the transcription factor E2F1, involved in apoptosis and cell proliferation which is consistent with our observations about the enrichment of mir-20 targets in this group of proteins. The other is TGFBR2 (transforming growth factor, beta receptor II), a member of the Ser/Thr protein kinase family that phosphorylates proteins regulating the transcription of genes related to cell proliferation. This is also consistent with our observations and may imply that this family of miRNAs is related to processes of protein modifications. The hsa-mir-106 target is RB1 (retinoblastoma 1) which has transcription coactivator activity and is involved in the negative regulation of cell growth.

Beyond their non-homogenous distribution, hsa-mir-367/-25/92 and hsa-mir-93/17-5p,/20/106 target sites occur in too many genes with quite different functions. So we suppose that here we have encountered not a known form of miRNA regulation (which is more individualized process), but, more likely, this is a part of unknown cellular signaling system. Before discussing some ideas about this in more details, we have to tell a little more about the other miRNA target sites.

#### B) Distribution of intermediate and rare target sites

Unlike for the widespread target sites, we can not understand much about the functions of these two groups of *Alu-*related sites from their distribution among the functional categories in our dataset. Anyway, some cases allowed us to make some plausible predictions.

Not surprisingly, the ‘development’ category is enriched in target sites for **let-7 group** of miRNAs. **Hsa-let-7b** occurs in this group 9 times **hsa-let-7c** – 7 times, and **hsa-let-7e** – 5 times.

**TarBase** reports also some function in oncogenesis and tumor suppression for **hsa-let-7b.** There is a strong support for this property in our set of genes. Besides KRAS and NRAS, the two validated oncogenes targets for hsa let-7b, four more cancer-related genes in our set have target sites for this miRNA: carcinoembryonic antigen-related cell adhesion molecule 8 (NM_001816), v-yes-1 Yamaguchi sarcoma viral oncogene homolog 1 (NM_005433), RAB21, member RAS oncogene family (NM_014999) and leucine zipper, putative tumor suppressor 1 (NM_021020). The last two genes have also target sites for hsa-let-7c, which implies combinatory action of these miRNAs

3 of 8 occurrences of **hsa-mir-143** also match the ‘development’ group. The validated target for this miRNA is mitogen-activated protein kinase 7. In response to extracelluar signals, this kinase translocates to cell nucleus, where it regulates gene expression by phosphorylating, and activating different transcription factors. Our data expands the picture and reveal additional complexity in it: 4 of ‘our’ targets of hsa-mir-143 are themselves transcription factors – forkhead box protein P4 (NM_001012426) and zinc finger proteins 490 (NM_020714), 526 (NM_133444) and 514 (NM_ 032788). Interestingly, zinc finger proteins contain hsa-mir-143 target sites outside their *Alu* insertions, while in the forkhead box protein it is in a sense *AluSp* insertion. This probably represents a typical example of expanding a miRNA-based regulatory network of a certain miRNA with a participation of an *Alu* element.

The predicted hsa-mir-452, for which there is no information in miRNA databases, has 5 of 12 occurrences in the ‘development’ group (of them, 2 in *Alu* insertions), so it is very probable that its function also is related to development.

Some miRNAs that are not localized in *Alu*s occur almost exclusively in the ‘development’ category: hsa-mir-199b (which validated target LAMC2 is involved in epidermis development), hsa-mir-149, -500, -512-5p -519b, -519e* and 527.

**Hsa-mir-510, -152 and -484** which occur predominantly in *Alu* insertions are enriched in the category ‘transcription regulation’; **hsa-mir-128b, -378 and -452** – in ‘cell cycle’. Here we meet again **hsa-mir-452**. Its miRNA site has two matches also in ‘transcription regulation’. Such appearance of a specific miRNA target site in three different but related categories is a clear indication for its function. Among the hsa-mir-452 targets are: inhibitor of growth family member 5 (NM_032329); zinc finger and SCAN domain containing 2 (NM_ 181877) with hsa-mir-452 target sites out of *Alu* insertions, and Mps One Binder kinase activator-like 2A (NM_130807) with target site in an anti-sense *AluYi6* insertion.

Besides the above mentioned hsa-mir-452, the ‘cell cycle’ category contains increased number of target sites for **hsa-let-7c, 7b, hsa-mir-128b, -214 and -378.** The cell cycle related function of **hsa-mir-378** is indirectly supported by a MiRanda prediction of target site for it – the centrosome associated actin homolog ARP1.

Other predictions, based on our dataset are:

- **hsa-mir-488** – very probably involved in apoptosis (2 of 3 occurrences, 1 of them in *Alu* insertion)
- hsa-mir-526b* - very probably involved in cell adhesion (2 of 3 occurrences)
- **hsa-mir-453 and hsa-mir-17-3p** (*Alu*-related) and **hsa-mir-22 and -302b** (not related to *Alus*) – are probably involved in transport
- **hsa-mir-17-3p, -412 and -453** – very probably targeting receptors (additional support: MiRanda-predicted target for **hsa-mir-453** – neuron derived orphan receptor 1)
- **hsa-mir-422a** – probably targeting structural proteins. (Indeed, MiRanda predictions for this miRNA are different: proteins involved in morphogenesis, transport and some protein kinases, but, in fact, one of its target in our set of genes unites all these different functions: neurofibromin 2 (NM_000268) – involved in negative regulation of cell proliferation and cell cycle, perception of sound, structural molecule activity (nucleus, cytosceleton))
- **hsa-mir-197** - very probably involved in immune response
- Two miRNAs (not related to *Alu*s in our dataset), **hsa-mir-412,** with almost 100% probability (4 of 4 occurrences), and **hsa-mir-193a** (4 of 6 occurrences) with a big probability are involved in signal transduction.

The category ‘metabolism’ is perhaps too broad to distinguish any clear possibility. There is some probability for **hsa-mir-526a** and **hsa-mir-211** to be involved in regulating metabolite processes, but more data are needed to make more clear predictions.

### *Alu* insertions in 3′UTRs as stress sensors for regulatory responses?

Not regarding their non-homogenous distribution, hsa-mir-367/-25/92 and hsa-mir-93/17-5p,/20/106 target sites occur in too many genes with quite different functions. Does this fact have any reason for the cell? Contemplating on this question guided us to a hypothesis for another, perhaps broader than miRNA regulation alone, scale of interactions.

In addition to *Alu* elements inserted in Pol II transcripts, a population of Pol III *Alu* transcripts is proved to exist in the cytoplasm of cells of almost all tissues [3, 38]. Two main forms of such *Alu* RNAs have been detected: full length *Alu* RNAs and small cytoplasmic *Alu* RNAs, including monomeric *Alu* sequences and some *Alu*-like sequences like BC200 gene (which is necessary for the function of neurons). All *Alu* sub-sub families are represented among cytoplasmic *Alu* RNAs, but the young ones predominate. Despite the high number of *Alu* copies in genomes, in normal conditions the cytoplasm of each cell typically contains less than 1000 copies of different *Alu* RNAs. The situation changes dramatically in cases of viral infection, alterations of nucleotide methylation status, heat shock and chemical manipulation. All these events cause accumulation of high levels *of Alu* RNAs in the cytoplasm [3, 39, 40]. Moreover, these *Alu* RNAs, like the 7SL RNA (from which they have originated about 60 million years ago) have retained their ability to bind SRP proteins and their homologs and to form SRP-like RNP particles [41, 42]. SRP function in eukaryotes is to target newly-translated proteins to membrane and/or to inhibit protein translation. On this base, some antiviral function of the *Alu* RNAs is proposed [43].

When we connected these facts to the data described in our analysis so far (many cases of *Alu* insertions in 3′ UTRs of genes, carrying potential miRNA target sites), an intriguing possibility emerged: in periods of cell stress, the antisense *Alu* insertions in 3′UTRs of mRNAs could interact complementarily and form RNA:RNA duplexes with the increased amount of sense *Alu* transcripts in the cytoplasm. This could have various and dramatic effects on miRNA- and other types of 3′UTR-localized regulation. For instance, in the case of miRNA regulation, the binding of such *Alu* RNA to the complementary *Alu* insertion in the 3′UTR could block the access of *Alu*-localized (and perhaps other) miRNAs and their miRNPs to their target sites. this could cause rapid increase in cellular concentration of many different proteins simultaneously. In this sense, it is also remarkable that, in our dataset, antisense *Alu* insertions and their miRNA target sites are enriched in the categories of proteins involved in ‘development’, ‘cell cycle’, ‘transport’, ‘transcription regulation’ and ‘immune response’. Moreover, antisense *Alu* inserts occur in all categories of proteins we investigated. Thus, the concentration of free *Alu* RNAs in the cytoplasm could play the role of a environment-sensitive, multi-state switch, flexible enough to adjust the level of miRNA regulation to requirements of the cell.

The expression of hsa-mir-367, -25, and 92 is experimentally validated in various cell types and tissues, including neurons and embryonic stem cells [29, 31, 45, 46]. Many of the proteins in our analysis are also expressed in embryo. This opens a perhaps very important link between various conditions and the response miRNA- and others of cellular regulatory network in development, and the mediator of these signals could be the increased level of *Alu* RNAs in cytoplasm, competing to miRNPs for connecting to the 3′UTRs. The possibilities for sensitive regulation that such system opens to the cells are almost unlimited. If we add to this picture the similar abilities of *Alu* elements inserted in 5′UTRs (not analyzed here), that gives another, even more direct ways for *Alu* elements to affect gene expression.

This *Alu*-mediated response system perhaps is not restricted to the antisense insertions and miRNA target sites they carry. In the case of sense (direct) *Alu* insertions, the SRP- and SRP-like proteins may be involved. They could bind the *Alu* elements inserted in 3′UTRs affecting this way the translation in various ways. In cases of increased amounts of *Alu* and *Alu-*like transcripts in cytoplasm (in times of stress), greater amount of such proteins could bind to them, leaving the 3′UTR-localized insertions uncovered. This, on its turn, could open some blocked miRNA target sites or other regulatory motifs or to trigger other types of regulation.

## Conclusions

As mentioned in the beginning of our article, our cells still live in an RNA-protein world. It seems that various, hypercomplex, epigenetic interactions underlie our life and evolution.

As we can conclude on the basis of our results, ***Alu*** **elements can serve as donors of miRNA target sites to various cellular genes**. We show that the *Alu* elements provide multiple potential miRNA target sites in the 3′UTRs of the analyzed cellular genes. Many of these genes are involved (or have become involved after the establishment of proper *Alu-*based target sites) in processes like regulation of transcription, cell cycle, cell proliferation, apoptosis, cell-cell contacts and signal transduction. These are key processes governing the organogenesis and development of complex eukaryotes, including humans.

But this is only the first plan of the picture. ***Alu*** **elements, carrying miRNA target sites, could interact on a system level with cytoplasmic** ***Alu*** **RNAs and/or proteins that bind them.** These interactions could depend on the amount of free cytoplasmic *Alu* RNAs, which on its turn depends of many factors as stress conditions, methylation, viral infections etc. Thus, the *Alu* insertions in UTRs and their ability to connect to free cytoplasmic *Alu* RNAs and/or 7SLRNA/Alu-binding proteins, may constitute **an unique cellular sensory system that could change dramatically or to fine-tune the gene expression according to the requirements of the internal or external environment of the cell.** This may have great effects on development, stress response, immunity and, ultimately, on the evolution of eukaryotic cells.

*Alu* elements are relatively evolutionary young, which makes the situation even more intriguing. The *Alu*-mediated interactions could be a new, recently formed and even still developing system of regulatory interconnections, ‘caught’ in a process of evolution. And as far as all of these interactions are primate-specific and many are human-specific, the *Alu-*mediated miRNA regulation could be an important explanation for the appearance of some primate-specific and human-specific traits.

As it becomes clear, the mobile elements interconnect practically all local and global systems of regulation of gene expression, and mediate their globalization and network support. The *Alu-*miRNA interaction, revealed originally in this study, brings a powerful support to the idea that mobile elements are universal interconnection link between cellular regulatory systems.

The mobile elements are universal agents of evolutionary change. The latest developments in the evolutionary ideas at molecular level are that evolution is a natural system engineering process. The natural genetic engineering has the potential to create hierarchical subsystems and complex networks of genome regulation [9]. Mobile elements, including *Alu*s are perhaps the most important tool of this natural engineering. So we will be glad if, in this article, we could set on its place even a single piece from the astonishing puzzle of the molecular evolution.

## Figures and Tables

**Table 1:** Some of the most abundant *Alu*-related miRNA target sites.

Target sites for:	Number of occurrences in 3′UTRs as a whole	Number of occurrences in Alu inserts	Alu orientation*
hsa-mir-20b	121	114	d
hsa-mir-17–5p	118	111	d
hsa-mir-20a	111	105	d
hsa-mir-106a	110	103	d
hsa-mir-92	103	99	c, rarely d
hsa-mir-93	107	99	d
hsa-mir-367	101	98	c, rarely d
hsa-mir-25	101	97	c, rarely d
hsa-let-7b	57	40	c, rarely d
hsa-mir-484	34	30	d
hsa-mir-453	37	30	c
hsa-let-7i	29	24	c&d
hsa-mir-346	23	15	d
hsa-let-7c	27	12	c&d
hsa-mir-106b	9	8	d
hsa-mir-422a	12	8	c&d
hsa-mir-520g	9	7	d
hsa-mir-452	12	7	c
hsa-mir-455	9	7	d
hsa-mir-372	8	7	d
hsa-let-7e	14	7	c
hsa-mir-187	12	7	c
hsa-mir-378	13	6	d
hsa-mir-197	9	5	d
hsa-mir-339	14	5	c&d
hsa-let-7g	7	5	c

* This column denotes the orientation of the Alu insert in which the target sites are localized: ‘d’ is for direct (sense) orientation, ‘c’ – for complementary (antisense) orientation of the *Alu* inserts.

**Table 2:** 14 main groups of proteins, defined on the base of most often occurring GOA keywords among the set of analyzed genes. The ratios ‘target sites/protein’ are shaded in yellow for total 3′UTRs and in light green for Alu inserts only. The 3 highest scores are shown in bold.

Keyword	No proteins	% of all proteins	Total content of miRNA target sites			Content of miRNA target sites in *Alu*s		

			MiRNAs, number	target sites, number	target sites/protein	MiRNAs, number	target sites, number	target sites/protein

Metabolism	41	17,23	90	326	7,95	24	198	4,83
Signal transduction	36	15,13	99	299	8,31	26	145	4,03
Transport	36	15,13	83	306	8,50	26	179	4,97
Regulation of transcription	31	13,03	98	298	**9,61**	29	158	5,10
Development	25	10,50	93	243	**9,72**	15	110	4,40
Immune response	23	9,66	68	183	7,96	18	106	4,61
Receptor	23	9,66	77	205	8,91	25	106	4,61
Nervous system	18	7,56	67	165	9,17	20	97	5,39
Cell cycle	16	6,72	56	147	**9,19**	25	93	**5,81**
Protein modification	15	6,30	59	121	8,07	20	67	4,47
Structural component	12	5,04	46	88	7,33	19	46	3,83
Apoptosis	11	4,62	53	100	9,09	15	53	4,82
Cell adhesion	11	4,62	42	90	8,18	20	64	**5,82**
Information processing	8	3,36	28	63	7,88	14	46	5,75
In all 238 proteins:			**153**	**1980**	**8,32**	**53**	**1095**	**4,60**
